# Cross-Domain Feature Enhancement-Based Password Guessing Method for Small Samples

**DOI:** 10.3390/e27070752

**Published:** 2025-07-15

**Authors:** Cheng Liu, Junrong Li, Xiheng Liu, Bo Li, Mengshu Hou, Wei Yu, Yujun Li, Wenjun Liu

**Affiliations:** 1 School of Computer Science and Engineering, University of Electronic Science and Technology of China, Chengdu 611731, China; 202112081309@std.uestc.edu.cn (C.L.); liyujun@uestc.edu.cn (Y.L.); 2 No. 30 Institute of CETC, Chengdu 610041, China; yuwei19@nudt.edu.cn; 3 National Key Laboratory of Security Communication, Chengdu 610041, China; 4 School of Computer and Software Engineering, Xihua University, Chengdu 610039, China; lijunrong@stu.xhu.edu.cn (J.L.); 3120210971107@stu.xhu.edu.cn (X.L.); libo@mail.xhu.edu.cn (B.L.); liuwenjun@mail.xhu.edu.cn (W.L.); 5 School of Big Data and Artificial Intelligence, Chengdu Technological University, Chengdu 611730, China

**Keywords:** password guessing, small samples, similarity computation, probabilistic context-free grammar

## Abstract

As a crucial component of account protection system evaluation and intrusion detection, the advancement of password guessing technology encounters challenges due to its reliance on password data. In password guessing research, there is a conflict between the traditional models’ need for large training samples and the limitations on accessing password data imposed by privacy protection regulations. Consequently, security researchers often struggle with the issue of having a very limited password set from which to guess. This paper introduces a small-sample password guessing technique that enhances cross-domain features. It analyzes the password set using probabilistic context-free grammar (PCFG) to create a list of password structure probabilities and a dictionary of password fragment probabilities, which are then used to generate a password set structure vector. The method calculates the cosine similarity between the small-sample password set *B* from the target area and publicly leaked password sets $A_{i}$ using the structure vector, identifying the set $A_{max}$ with the highest similarity. This set is then utilized as a training set, where the features of the small-sample password set are enhanced by modifying the structure vectors of the training set. The enhanced training set is subsequently employed for PCFG password generation. The paper uses hit rate as the evaluation metric, and Experiment I reveals that the similarity between *B* and $A_{i}$ can be reliably measured when the size of *B* exceeds 150. Experiment II confirms the hypothesis that a higher similarity between $A_{i}$ and *B* leads to a greater hit rate of $A_{i}$ on the test set of *B*, with potential improvements of up to 32% compared to training with *B* alone. Experiment III demonstrates that after enhancing the features of $A_{max}$ , the hit rate for the small-sample password set can increase by as much as 10.52% compared to previous results. This method offers a viable solution for small-sample password guessing without requiring prior knowledge.

## 1. Introduction

As a fundamental mechanism for ensuring user account security, research on password guessing techniques is of great practical significance for evaluating the effectiveness of account protection measures and conducting system penetration testing. Based on the type of data required, password guessing techniques can generally be categorized into three types: training-free, intra-site, and cross-site [1]. Among these, the latter two belong to data-driven password guessing methods [2,3,4,5], which typically require large-scale training datasets and often rely on probabilistic or machine learning models [6] to generate guessed passwords. Studies show that due to legal constraints from data privacy regulations and technical challenges in data acquisition, security researchers often only have access to limited, unidentified publicly leaked password datasets or small-scale target password sets. This limitation poses a bottleneck for traditional data-driven password guessing methods that heavily depend on large sample sizes.

In recent years, academic research on password guessing has made significant progress, particularly in data-driven approaches. Among them, intra-site password guessing has attracted the most theoretical and practical attention [7], as it allows for the extraction of password features more closely aligned with the target system, potentially leading to higher hit rates. However, with the strengthening of cybersecurity measures and the enforcement of data privacy laws, acquiring sufficiently large password datasets in intra-site scenarios has become increasingly difficult. Consequently, researchers often face a dilemma: the size of the available training dataset for password guessing is extremely small, usually ranging from dozens to hundreds of samples—far below the typical requirement of over $10^{4}$ samples needed by traditional data-driven approaches. Therefore, it is of considerable practical importance to conduct in-depth research on password guessing techniques applicable to small-sample scenarios, especially within the intra-site context, to address the challenges posed by the scarcity of password data in real-world applications. Currently, methods for small-sample password guessing can be primarily classified into two categories:

1. Enhancing the corpus: To address sample limitations, additional personal data, topics, regional corpora, password fragments, and other relevant information can be incorporated. In 2016, Wang et al. [8] introduced a targeted online password guessing framework based on user personal information, which combines seven probabilistic attack models to enhance guessing efficiency in scenarios with limited samples. In 2019, Bi et al. [9] developed a topic-based PCFG model that integrates user interests, cultural backgrounds, and other topic-related data to lessen dependence on leaked password information. In 2021, Gan et al. [10] introduced a corpus-based password guessing approach that leverages extensive corpora to make up for the scarcity of words in smaller training sets, thus enhancing password guessing efficiency in such contexts. In 2022, Chen [11] suggested generating guessing rules that can be adapted for longer passwords by examining the corpus transfer characteristics of shorter ones. That same year, Han et al. [12] tackled the challenge of traditional methods that depend on large datasets by proposing a parameterized hybrid guessing framework (hyPassGu). This framework merges the strengths of various techniques, including probabilistic context-free grammar (PCFG) and Markov models, along with model pruning and optimal guessing number allocation strategies to create effective guessing sets with minimal data. However, while these methods can produce relatively comprehensive password guessing dictionaries, they are heavily dependent on corpus information, and research shows that acquiring relevant corpus data can be difficult in real-world applications.
2. Feature extraction approach: This method involves using AI models to identify additional potential features to make up for a lack of samples. Significant advancements have been made in neural password models. For instance, PassGAN [13] was the first to apply generative adversarial networks to understand the underlying distribution of passwords. Following this, models like FLA [14] and Transformer-based approaches [15], including discussions around PassGPT, have been able to capture intricate long-range dependencies in passwords through more advanced architectures. In 2022, Geng [16] introduced a small-sample password guessing model that employs multi-task learning, which reduces the dependence on a single large sample set by sharing feature representations across different datasets. Additionally, transfer learning techniques have emerged as a crucial strategy for tackling the small-sample issue.Melicher et al. [17] were pioneers in showing that pre-training recurrent neural networks (RNNs) or gated recurrent units (GRUs) on extensive leaked datasets allows the general knowledge of password structures to be transferred and adapted to new, smaller contexts. In 2023, Wang et al. [18] introduced the Pass2Edit algorithm, which utilizes GRU to understand the effects of password modification actions. With just one previous password from the user, it can produce a high-hit-rate guessing sequence, achieving a 24.2% hit rate for regular users within 100 attempts. In the same year, Wei [19] employed a pre-trained model to extract general features from a large leaked dataset and apply them to a small-sample context, enhancing the model’s adaptability to specific user demographics, while feature extraction methods can offer robust solutions for various complex issues, the high training costs and low efficiency they involve present significant challenges that researchers and developers need to tackle.

In this research, we present a novel approach to password guessing that leverages cross-domain feature enhancement, specifically designed to address the challenge of low guessing accuracy associated with small-sample password sets, denoted as *B*, within the target domain. The experimental framework utilizes publicly available leaked password datasets, which include information from platforms such as Youku and Gmail, among others. We define the collection of publicly leaked passwords as $A=\{A_{1},A_{2},\ldots ,A_{n}\}$ , where *A* represents the comprehensive set of all publicly leaked password datasets, and $A_{i}$ signifies the *i*-th independent dataset within this collection. To enhance the guessing accuracy, we compute the cosine similarity between the target password set *B* and each dataset $A_{i}$ , subsequently identifying the dataset $A_{max}$ that exhibits the highest similarity. This dataset is then utilized for feature enhancement, resulting in a password set that demonstrates an improved hit rate for the target domain. The key innovations of this methodology are as follows:

1. This study introduces a methodology for evaluating the similarity of password sets through the structural probability vectorization of these sets, enabling a quantitative analysis of the degree of similarity among various password collections. The findings indicate that this approach can reliably quantify the similarity between a limited sample of password sets within a specific domain and a publicly available compromised set, provided that the size of the set exceeds 150;
2. This study validates the hypothesis that a greater degree of similarity between $A_{i}$ and *B* correlates with an increased hit rate of $A_{i}$ on the test set associated with *B*. Experimental results indicate that the hit rate for password guessing is enhanced by as much as 32% compared to training conducted with *B*;
3. The impact of the guessing hit rate is particularly pronounced when there is a low degree of similarity between $A_{i}$ and *B*. Empirical findings indicate that when the similarity index between $A_{i}$ and *B* falls below 0.6, the application of this method can enhance the guessing hit rate by a maximum of 10.52%;
4. Our approach leverages password set similarity to transfer the most relevant features from publicly leaked datasets to the intra-site small-sample password set. This effectively addresses the issue of insufficient feature extraction in traditional intra-site methods under small-sample conditions, thereby significantly improving the password guessing hit rate in a simple yet efficient manner.

## 2. Background Knowledge

### 2.1. Probabilistic Context-Free Grammar

In this study, we employ the probabilistic context-free grammar (PCFG)-based password guessing model introduced by Weir et al. in 2009 to train the parameters *B* and $A_{i}$ . This model generates a compilation of probabilities associated with password structures and a lexicon of probabilities for password fragments by examining a substantial dataset of actual password collections, thereby facilitating the efficient guessing of passwords. The entire methodology is delineated into two distinct phases:

During the training phase, the composition of each password is systematically analyzed by segmenting it into three distinct components: the letter segment (L), the number segment (D), and the special character segment (S). Each segment is extracted and subsequently labeled based on its respective length. For instance, the password “password12345!!!” can be delineated into a letter segment of length 8, a number segment of length 5, and a special character segment of length 3, resulting in the structural label L8D5S3. Similarly, the structure of a passphrase can also be represented as L8D5S3. The structural probability is then computed based on the frequency of occurrence of this structure within a dataset of passphrases, leading to the generation of a probability list for the passphrase structures. Concurrently, for each password structure, the frequency of occurrence of each structural fragment within the password dataset is quantified, and its probability is derived from this frequency, culminating in the creation of a password fragment probability dictionary. This comprehensive, stepwise methodology facilitates the effective training of the model on the password dataset.

In the initial phase of the process, the starting symbol of the probabilistic context-free grammar (PCFG) is designated as the initial node of the search. Subsequently, new nodes, representing new password strings, are generated through the iterative application of the generative rules of the PCFG. This process prioritizes password rules with higher probabilities to ensure that the generated passwords more accurately reflect the actual distribution of existing passwords. The specific probability calculation is expressed as follows: P(password12345!!!)=P(L8D5S3)×P(L8→Password)×P(D5→12345)×P(S3→!!!)=p . The resulting probability *p* indicates the frequency of password generation. Furthermore, this study establishes a minimum probability threshold for password generation to prevent infinite loops in the generation process and to avoid the creation of excessively long password strings, which could lead to inefficient use of computational resources and a decrease in generation efficiency.

### 2.2. Cosine Similarity

Cosine similarity is a quantitative metric utilized to assess the degree of similarity between two vectors. This measure is derived by calculating the cosine of the angle formed between the two vectors. The resulting value ranges from −1 to 1, where a value approaching 1 indicates a higher degree of similarity between the vectors.

The mathematical expression for cosine similarity is as follows:

(1) $$CosineSimilarity\left(A,B\right)=\frac{A\cdot B}{∥A∥∥B∥}=\frac{\sum_{i=1}^{n}A_{i}\cdot B_{i}}{\sqrt{\sum_{i=1}^{n}A_{i}^{2}}\cdot \sqrt{\sum_{i=1}^{n}B_{i}^{2}}}$$

Included among these are:

- *A* and *B* represent two vectors for which the computation of similarity is to be conducted, respectively;
-
$A_{i}$ and $B_{i}$ represent the ith eigenvalues associated with their respective eigenvectors;
- The notation ∥A∥ and ∥B∥ represents the Euclidean norms of the vectors *A* and *B*, respectively.

## 3. Algorithmic Progression

**Figure 1:**
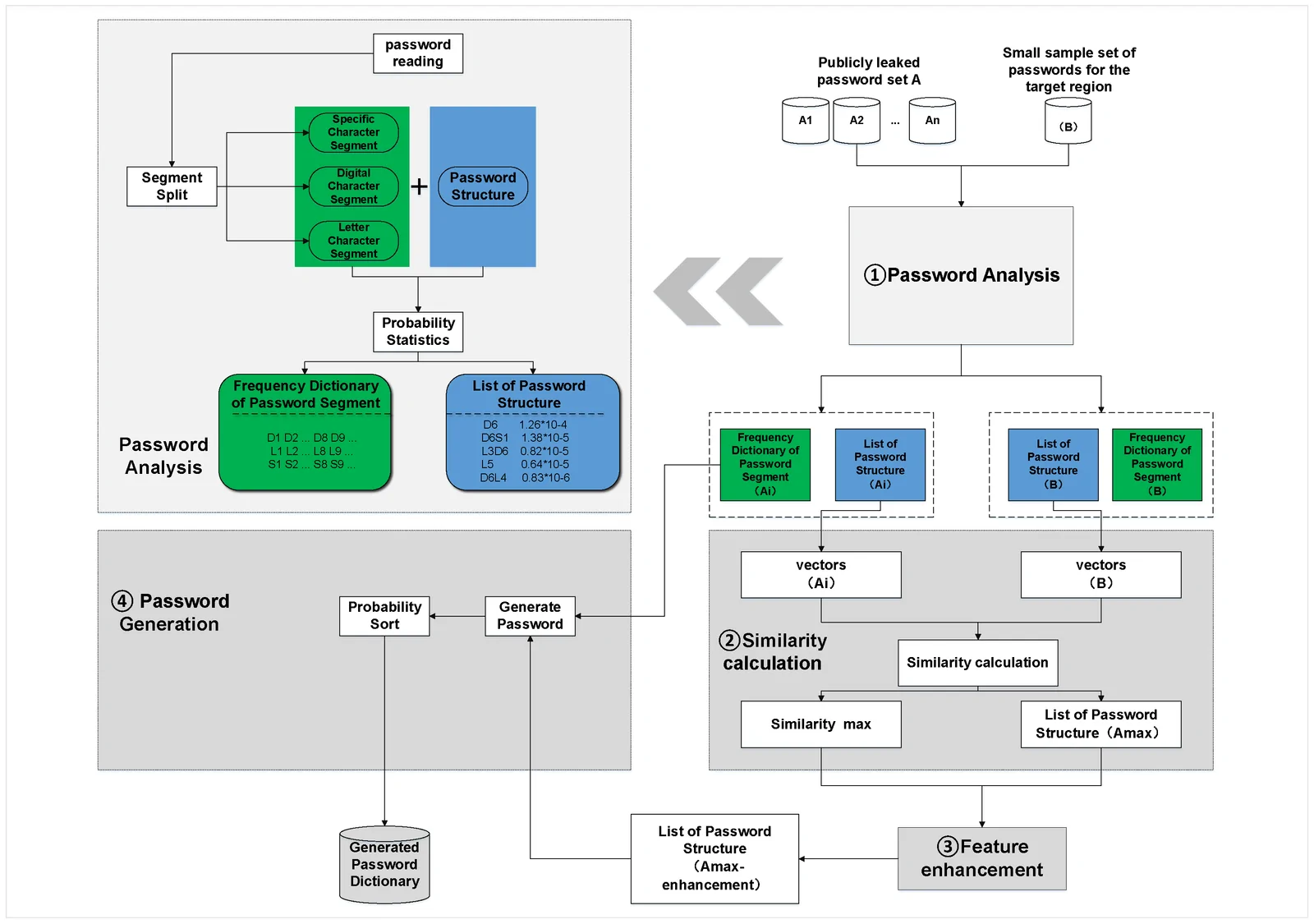
Schematic representation of a small-sample password guessing technique utilizing cross-domain feature enhancement.

The limited availability of small-sample features within the target domain induces distributional bias, resulting in a generalization bottleneck in the generative model. This bottleneck is characterized by insufficient coverage of the password space and a low hit rate. To address this issue, we propose a small-sample password guessing methodology that leverages cross-domain feature enhancement, which is predicated on three key assumptions (refer to Figure 1 for the schematic representation). The efficacy of this approach has been validated. The three assumptions are as follows:

1. The password set $A_{i}$ , which bears resemblance to *B*, has the capacity to significantly enhance the password characteristics associated with *B*.
2. The pertinence of password features to the target region can be improved by enhancing the relevant features of *B* in $A_{i}$ ;
3. Assumption 1 can expand the features, and Assumption 2 can enhance the features, and the combination of the two can improve the hit rate of password guessing in the target region.

This approach comprises four distinct stages:

(1) Conduct a statistical analysis of the password dataset utilizing the probabilistic context-free grammar (PCFG) methodology. This analysis should yield a probability list detailing the structural components of the passwords, as well as a probability dictionary for the fragments within the password set;
(2) Similarity computation involves converting the probability list of password structures from a given password set into a corresponding structure vector. Subsequently, the cosine similarity is calculated between the structure vectors of the password sets, serving as a measure of similarity between these sets. The objective is to identify the publicly leaked password set, denoted as $A_{max}$ , that exhibits the highest degree of similarity;
(3) Feature enhancement: Enhance the structure of *B* in the password structure probability list of $A_{max}$ and the fragment of *B* in the password fragment probability dictionary.
(4) Password generation: generate a guess password set based on the password structure probability list and password fragment probability dictionary of $A_{max}$ after feature enhancement.

### 3.1. Password Analysis

This study conducts a comprehensive statistical analysis of the variable *B* utilizing the probabilistic context-free grammar (PCFG) methodology. Specifically, we parse and generalize a substantial number of samples from the password dataset to extract the structural features of the passwords and the probability distributions of their components. Based on this analysis, we derive two significant statistical outcomes. The first is a probability list of password structures, which delineates the length and composition of each field within a password, encapsulating the primary feature information. This list meticulously documents the frequency of various password structures alongside their corresponding probability values. The second outcome is a probability dictionary of password fragments, which encompasses common segments of passwords, including combinations of letters, numbers, and special characters. The structure of the passphrase set refers to the structural characteristics inherent to all passphrases within a given set. By examining the structure of the password set, we gain enhanced insights into the similarities and differences that exist among various password sets.

### 3.2. Similarity Calculation

We assess the structural cosine similarity among the password sets to identify the publicly leaked password set $A_{max}$ within $A_{i}$ that exhibits the greatest similarity to *B*. This approach aims to ensure that $A_{max}$ not only encompasses the characteristics of *B* more comprehensively under the prevailing conditions but also compensates for the deficiencies in the password set *B*. The subsequent steps outline the detailed methodology for calculating the structural similarity.

- First, the structural characteristics are derived for each password set $A_{i}$ within the collections *B* and *A* through the process of model training, resulting in the generation of a structural probability list for each password set;
- Then, the structural features of a password set $A_{i}$ in *A*, which is to be measured for structural similarity, are concatenated with the structural features of *B* to get the length of the proposed vector and the meaning of each component.
- The structural probabilities of $A_{i}$ and *B* are subsequently incorporated into the vector file in accordance with the sequential interpretations of the components. Ultimately, this process yields the structural feature vectors associated with $A_{i}$ and *B*.
- Ultimately, the cosine similarity is computed for the derived vectors utilizing the formula outlined in Section 2.2, in order to assess the structural similarity between $A_{i}$ and *B*. The vector $A_{i}$ exhibiting the highest degree of similarity is designated as $A_{max}$ .

### 3.3. Feature Enhancement

During the course of our experiments, we observed that when the structural similarity value falls below a specified threshold (refer to Section 4 for the detailed methodology), the structural characteristics of $A_{max}$ can be enhanced by incorporating the structural features of *B*. This integration compensates for the deficiencies in $A_{max}$ due to the absence of *B*’s features, thereby increasing the accuracy of predicting *B*. The augmentation technique involves enhancing the probabilities of the structures and fragments present in *B* within $A_{max}$ . The comprehensive algorithmic procedure is as follows Algorithm 1:
**Algorithm 1:** Procedure for Enhancing Structural Features  **Input**: 
$P_{a}$
: Probability distribution of 
$A_{max}$
; 
$P_{b}$
: Probability distribution of B; 
α
:     Enhancement strength parameter  **Output**: 
$P_{E}$
: Enhanced probability distribution
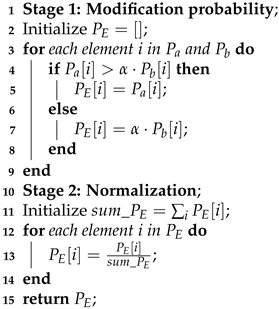


In this context, let $P_{a}$ and $P_{b}$ denote the probabilities associated with the respective structures or elements derived from training the model on $A_{max}$ and *B*. The parameter α serves as a customizable factor that modulates the influence of the features of *B* to augment those of $A_{max}$ . During the enhancement procedure, if $P_{a}>\alpha \cdot P_{b}$ , the probability value $P_{a}$ for $A_{max}$ remains unchanged, as it is indicative of a more prevalent usage pattern at that juncture. Conversely, when $P_{a}<\alpha \cdot P_{b}$ , the probability for $A_{max}$ is adjusted to $\alpha \cdot P_{b}$ , thereby aligning $A_{max}$ more closely with the characteristics of *B*. Following the completion of the probability enhancement, a normalization process is conducted to ensure that the aggregate of the probabilities for all structures and elements equals 1, thereby validating the reasonableness and integrity of the probability distribution.

### 3.4. Password Generation

After constructing the model for feature enhancement, we proceed to the password generation phase. Beginning with the initial symbols and utilizing the probabilistic dictionary of password structures, we prioritize the structures with the highest probabilities for expansion. Using the dictionary of password fragment probabilities, we populate the password structure by emphasizing the fragments with the highest likelihood. In this way, the initial symbol is gradually expanded into a complete string.

After generating the string, the resulting passphrase is evaluated to determine whether it meets the predefined length requirements and possesses adequate complexity. For instance, it should include a minimum number of different character types, such as uppercase letters, lowercase letters, numbers, and special symbols. If the generated passphrase fails to satisfy these criteria, the system will backtrack to the previous generation step and create a new segment of the passphrase by re-selecting alternative generative rules based on their probability until a passphrase that fulfills all requirements is produced.

## 4. Experiments and Analysis of Results

In order to systematically evaluate the effectiveness of the method proposed in this paper, we have constructed a three-level validation system. This system assesses the method’s effectiveness from three dimensions: data constraints assessment, core assumptions, and effect analysis following feature enhancement.

1. Data Constraint Evaluation: Assess the data constraints for similarity metrics and quantitatively establish the minimum sample size threshold required for stable similarity metrics within a small-sample password set *B*;
2. The core hypothesis is to verify whether an increase in similarity between $A_{i}$ and *B* corresponds with an increase in the guessing hit rate of the training-generated password set, thereby confirming the positive correlation between similarity and hit rate.
3. Analyzing the Impact of Feature Enhancement: This study examines the varying effects of feature enhancement on different sets of similar passwords.

Based on the research questions outlined above, we designed and conducted the following three experiments.

### 4.1. Data Sets

Since 2009, several large-scale data leakage incidents have come to light [6]. In 2011, a number of well-known domestic Internet companies, including Tianya, experienced user password leakage incidents that resulted in the exposure of personal information for approximately 40 million users [20]. In 2014, 12306, a prominent ticketing platform, was also affected, with leaked data involving users’ accounts, plaintext passwords, ID numbers, and private information such as email addresses [21]. In 2015, Twitch, a live gaming platform, suffered a massive data breach in which the password files of about 55 million users were compromised, potentially triggering the leakage of accounts related to Gmail and other services [22]. In 2016, security researchers uncovered a significant data breach involving approximately 9.86 GB of Twitter user datasets, which contained more than 200 million records linked to user account profiles, names, and email addresses [23]. In 2017, CashCrate experienced a data breach that compromised information including users’ email addresses, names, passwords, and physical addresses [24]. These ongoing data leakage events provide valuable data support for password guessing research and serve as a practical basis for optimizing guessing models.

Based on password databases derived from public leaks, the dataset utilized in this paper is divided into two parts: the small-sample password set *B* from the target region and the publicly leaked password clusters $A=A_{1},A_{2},...,A_{n}$ . Here, *A* represents all publicly leaked password sets within the complete dataset, while $A_{i}$ denotes the *i*-th independent publicly leaked password set. The set *B* is further divided into two subsets, B_CN and B_EN , represented by “12306” and “cashcrate,” respectively, reflecting the characteristics of the samples in two distinct environments. To investigate the effect of training set size on model performance, we sampled B_CN and B_EN hierarchically, gradually reducing the training set size from 500 to 10 entries. Correspondingly, the size of the test set was gradually increased from 69,500 to 69,990 entries. The publicly leaked passphrase set $A_{i}$ comprises six categories of data, each with a sample size of 70,000, covering several common service platforms and tools, such as Tianya, Gmail, Twitter, and 126, among others. This dataset provides rich diversity and reflects real-world password usage patterns for experimentation (See Table 1).

### 4.2. Evaluation Indicators

In this paper, the *Hit Rate* (HR) is utilized as the primary metric for assessing the performance of the model. The hit rate is defined as the proportion of generated password guessing dictionaries that include the test password. The formula for calculating the hit rate is as follows:

(2) $$Hit\;Rate=\frac{N_{hit}}{N_{title}}$$

Among them:

-
$N_{hit}$ indicates the number of test passphrases included in the generated passphrase guessing dictionary.
-
$N_{title}$ indicates the total number of test passwords.

### 4.3. Experiment 1: Evaluation of Data Constraints for Similarity Metrics

In the predefined context of this paper, a smaller sample from the password set is utilized, meaning that the number of passwords included is limited. However, when the sample size of the passwords to be guessed is too small, its similarity to other password sets becomes highly unstable. To ensure the robustness of the similarity metric, this experiment assesses the data constraints of the similarity metric to identify the minimum effective sample size, denoted as *B*. This involves determining a threshold value for the minimum number of passwords that can reliably reflect its similarity with other password sets. By establishing this threshold, a dependable benchmark for password set similarity metrics is created, which not only meets the preconditions of the experiment but also provides a foundation for future research.

**Figure 2:**
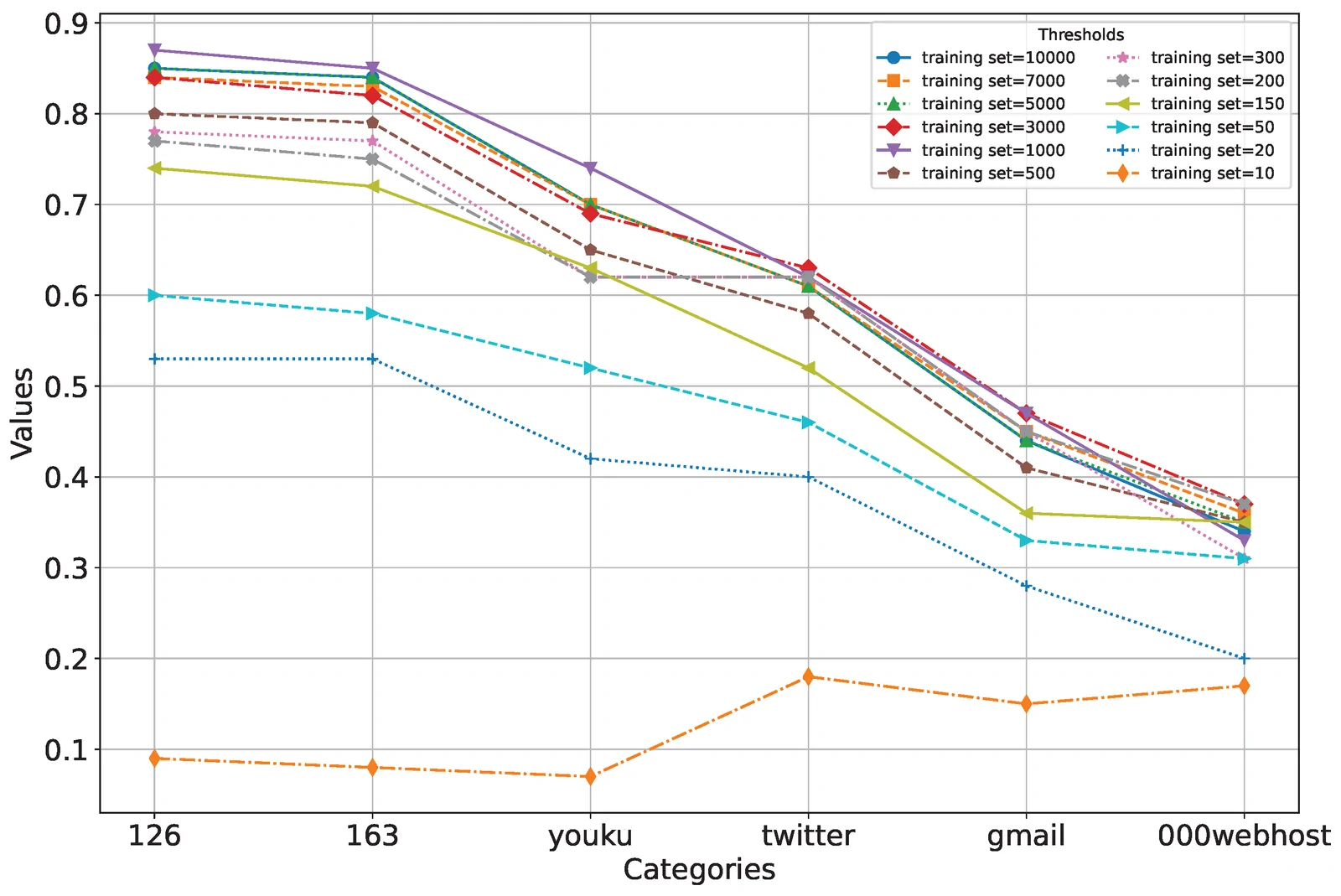
Similarity case between the password set B_CN and $A_{i}$ .

**Figure 3:**
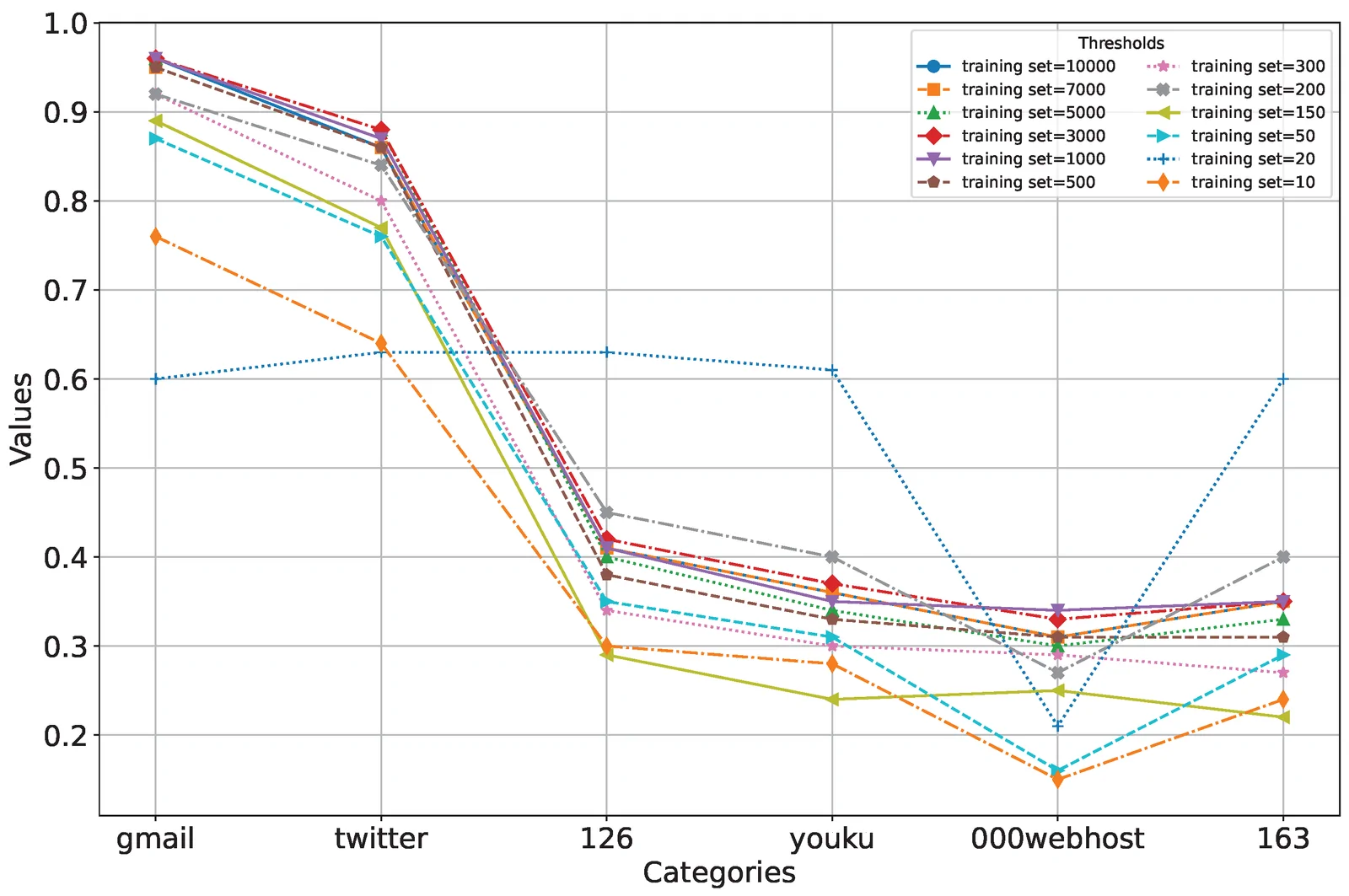
Similarity case of password set B_EN with $A_{i}$ .

For this reason, we divided the experiments into Chinese and English environments, corresponding to the password sets B_CN and B_EN , respectively. We conducted twelve sets of sampling experiments with training set sizes based on these two small-sample password sets, with the sizes of the training sets being 10,000, 7000, 5000, 3000, 1000, 500, 300, 200, 150, 50, 20, and 10, in that order. In each set of experiments, the similarity of *B* to $A_{i}$ is calculated separately. The specific steps are as follows: First, merge *B* and $A_{i}$ pairwise to form a concatenated set and generate the corresponding vector files. Then, perform cosine similarity computations on each set of vectors to obtain the similarity between the twelve sets of B_CN and $A_{i}$ in the Chinese environment, as well as the similarity between the twelve sets of B_EN and $A_{i}$ in the English environment (See Figure 2 and Figure 3).

The experimental results demonstrate the similarity between the password sets B_CN and B_EN in relation to $A_{i}$ within Chinese and English environments, respectively, across varying sample sizes. The horizontal axis of the graphs denotes the categories of password sets, while the vertical axis indicates the similarity values. The twelve sets of lines represent different training set sizes.

As a whole, the fluctuation in similarity between *B* and $A_{i}$ gradually increases as the size of the training set decreases. This fluctuation is particularly significant when the sample size is fewer than 50 entries. In the Chinese context, the similarity between the password set B_CN and the password sets “126”, “163”, and “tianya” in *A* is notably abnormal when the number of samples is 50. Similarly, in the English context, the password set B_EN exhibits anomalous fluctuations in similarity to the password sets “000webhost”, “126”, “163”, and “tianya” at a sample size of 50. Given the premise of small sample sizes and the need for stability in similarity, it is more appropriate to select 150 as the threshold for the small-sample training set in the next experiment. A sample size below this threshold may result in unstable experimental outcomes due to significant fluctuations in similarity, while a sample size above this threshold may not fulfill the requirements for small-sample experiments.

### 4.4. Experiment 2: Verifying the Positive Correlation Between Similarity and Hit Rate

In order to verify the proposed positive correlation between similarity and hit rate, this experiment is conducted in both a Chinese environment ( $B_{CN}$ ) and an English environment ( $B_{EN}$ ). The goal is to identify the leaked password set $A_{max}$ that is most similar to the small sample *B* (which includes both $B_{CN}$ and $B_{EN}$ ) and to assess its effectiveness in enhancing password guessing accuracy within small samples. (See Table 2).

The experiment builds upon the findings of Experiment 1, establishing that the minimum stable number of entries for *B* (which includes both B_CN and B_EN ) is 150. Under these conditions, the training set for B_CN ( B_CN_training ) consists of 150 entries, while the test set for B_CN ( B_CN_test ) comprises 69,850 entries. Similarly, the training set for B_EN ( B_EN_training ) also contains 150 entries, and the test set for B_EN ( B_EN_test ) includes 69,850 entries.

First, we will calculate the cosine similarity between B_CN_training and each of the mnemonic sets $A_{1}$ through $A_{6}$ to obtain the similarity values between B_CN_training and $A_{1}$ to $A_{6}$ . The same method will be applied to the datasets of B_EN_training with $A_{1}$ to $A_{6}$ to compute the similarity results in the English context. By comparing these similarity values, we can rank the similarity between each $A_{i}$ and *B*, allowing us to filter the leaked password set $A_{max}$ that is most similar to *B*.

**Figure 4:**
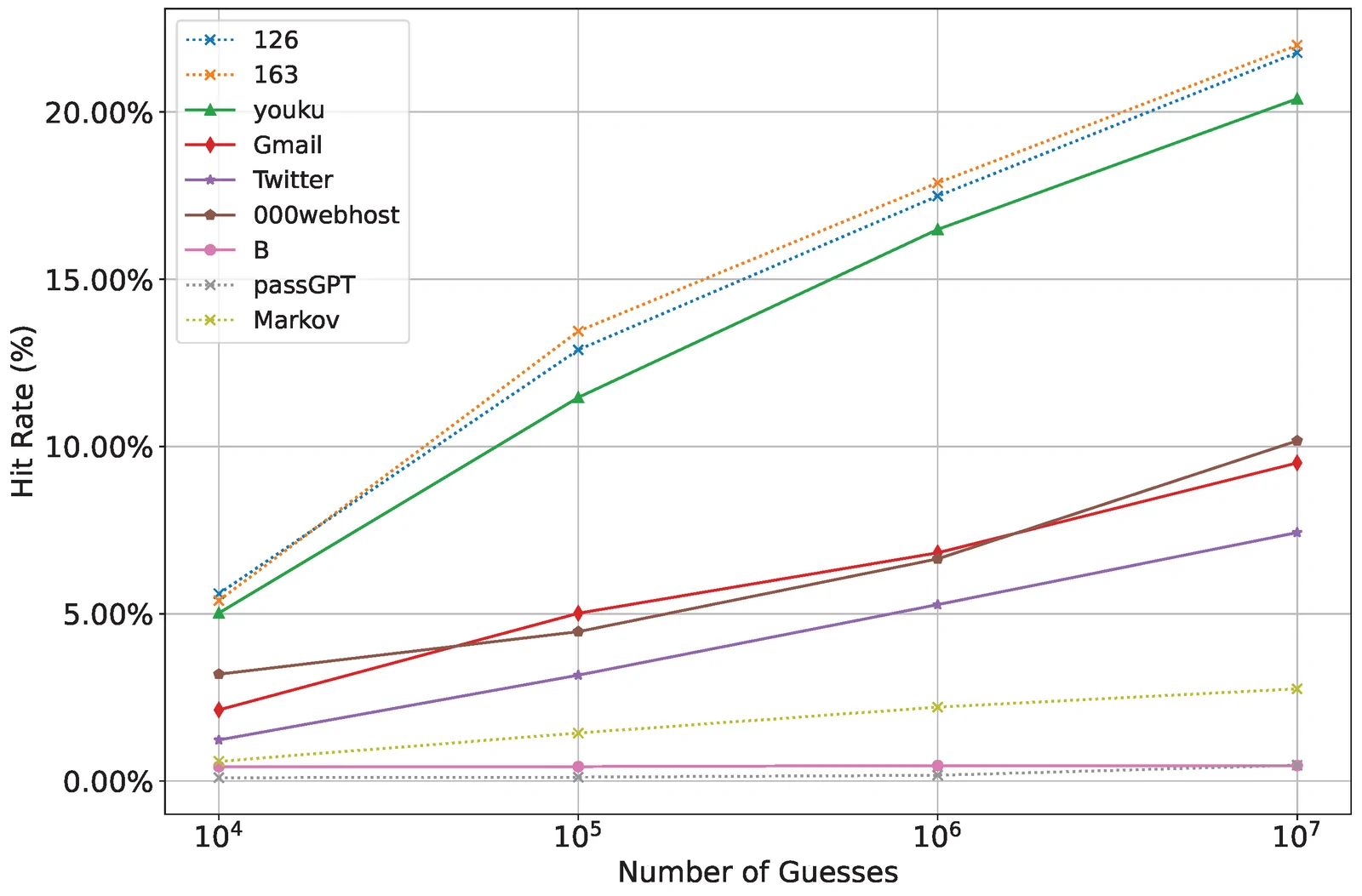
Hit rate of B_CN_test in Chinese environment.

**Figure 5:**
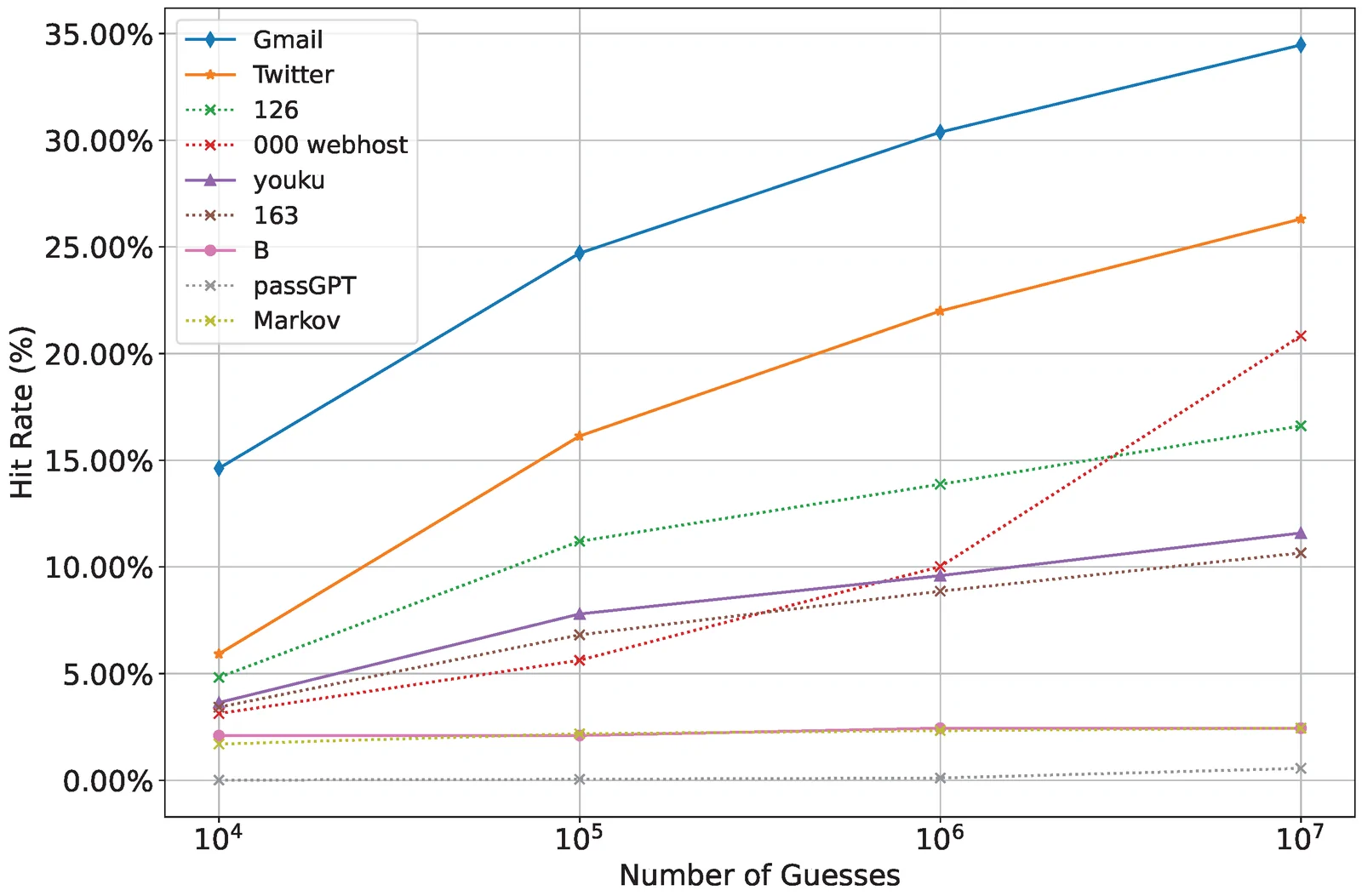
Hit rate of B_EN_test in English environment.

We begin with the most similar leaked password set, organized by similarity, and train it using the traditional probabilistic context-free grammar (PCFG) method in a Chinese environment. The dataset, denoted as B_CN_training , is trained with all six sets of $A_{i}$ using the traditional PCFG approach. Corresponding dictionaries are generated sequentially, and guesses are produced based on these dictionaries according to breadth-first search (BFS) rules.To compare performance, we also introduced PassGPT and Markov models as control groups. Simultaneously, we impose a maximum depth limit, allowing for the generation of guesses in sets of $10^{4}$ , $10^{5}$ , $10^{6}$ and $10^{7}$ . The generated guesses from each group are then matched against B_CN_test to evaluate the hit rate. A similar process and flow are applied in the English environment, and the results are as follows Figure 4 and Figure 5:

These two charts illustrate the hit rate of B_CN_test in a Chinese environment and B_EN_test in an English environment, respectively. The horizontal axis of the chart represents the number of guesses, which are categorized in orders of $10^{4}$ , $10^{5}$ , $10^{6}$ , and $10^{7}$ . The vertical axis indicates the hit rate as a percentage. The six sets of lines represent the hit rates of six different password sets under varying numbers of guesses.

Through in-depth observation and analysis of the experimental results, a clear law emerges: whether in a Chinese or English environment, the greater the similarity of the password set, the higher its hit rate for B_CN_test and B_EN_test . For instance, in the charts for the Chinese environment, the lines corresponding to the 126 and 163 password sets generally exhibit higher hit rates than the other datasets for the same number of guesses. Similarly, in the charts for the English environment, the lines corresponding to the Gmail dataset also demonstrate more pronounced hit rates for the respective number of guesses. This result strongly supports our initial conjecture that if $A_{i}$ is more similar to *B*, then $A_{i}$ will have a higher hit rate on the *B* test set.

Further in-depth exploration reveals that the most similar set of passphrases excels in terms of hit rate and can significantly enhance the hit rate, typically ranging from 5% to 32%. For instance, in the case of the Gmail dataset in English, as the number of guesses increases from $10^{4}$ to $10^{7}$ , the hit rate rises from approximately 15% to about 35%, representing a substantial increase. This serves as strong evidence for the validity and accuracy of our method, demonstrating that the most similar password set, $A_{max}$ , effectively improves the hit rate of guessing for small samples.

### 4.5. Experiment 3: Feature Enhancement and Effect Analysis

In order to further improve the hit rate, we will perform feature enhancement on $A_{i}$ . We anticipate that this method of feature enhancement and probability adjustment will enable $A_{i}$ to better capture the unique characteristics of *B*. Consequently, this will allow for the more accurate generation of passwords that align with the patterns of *B* during password guessing, thereby improving the hit rate and achieving optimal guessing results.

**Figure 6:**
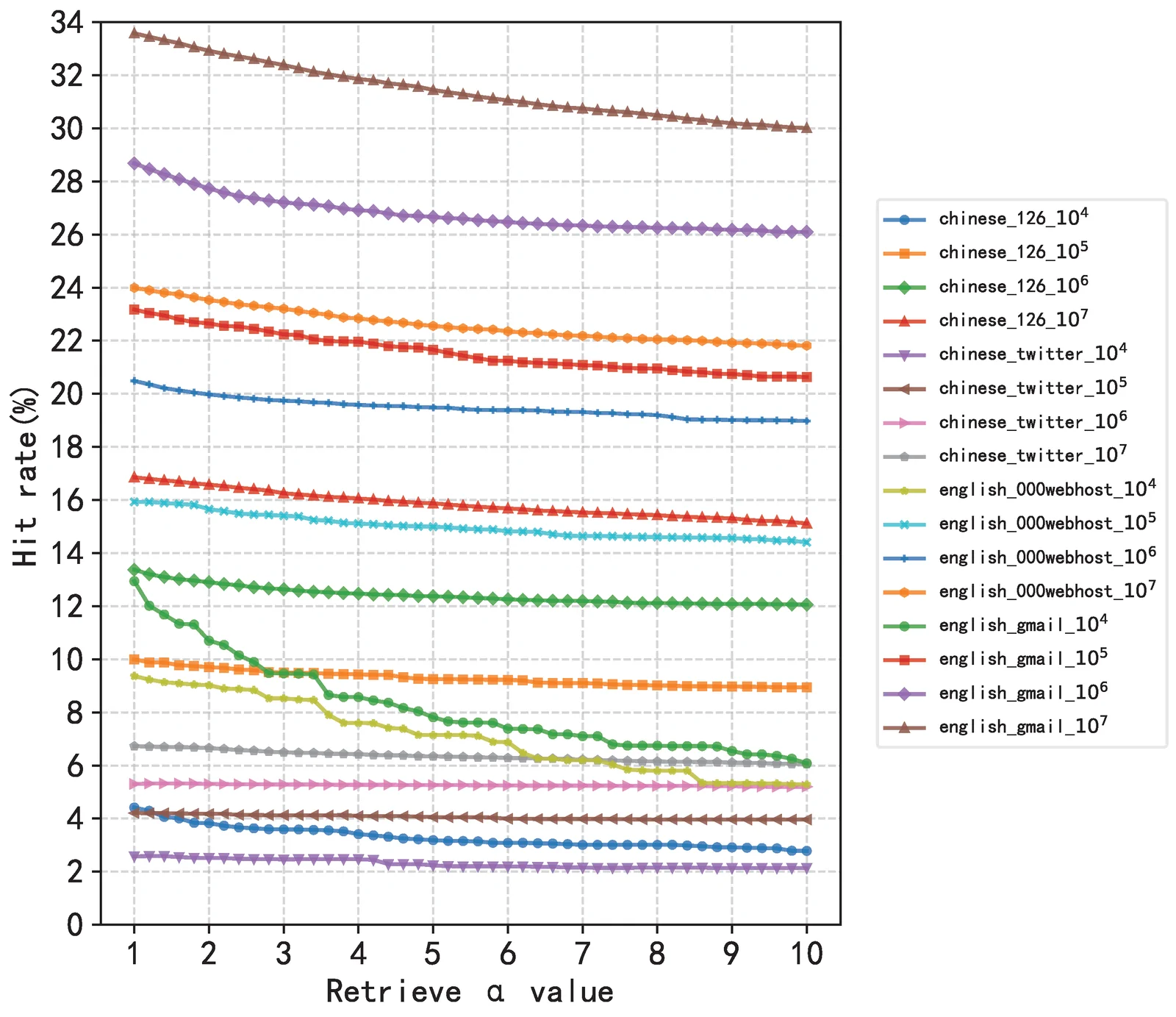
Comparison of hit rates for adjusting parameter α .

According to the conclusion of Experiment 1, we use the dataset *B*, which consists of the 150 samples from Experiment 1, and the features are enhanced for the training results in the Chinese environment (B_CN) and the English environment (B_EN). Then, the strength of feature enhancement of $A_{i}$ by the features of *B* is adjusted by introducing a custom parameter α (which cannot be less than 1), and the list of password structures of $A_{i}$ and the probability dictionary of each password fragment are modified accordingly. In order to systematically evaluate the effect of feature enhancement, we introduce the parameter α and adjust it dynamically, taking values from 1 to 10 with a step size of 0.2, to quantitatively analyze the effect of enhancement strength on model performance. We take the 126 and Twitter password sets as examples in the Chinese environment, and Gmail and 000webhost password sets as examples in the English environment, and the experimental results are as follows Figure 6:

From the experimental results, it is evident that the system’s hit rate exhibits a monotonically decreasing trend as the value of α increases. Based on the findings of this empirical analysis, we will fix the value of α at 1 in the subsequent feature enhancement process to achieve a better balance between the enhancement effect and system performance.

**Figure 7:**
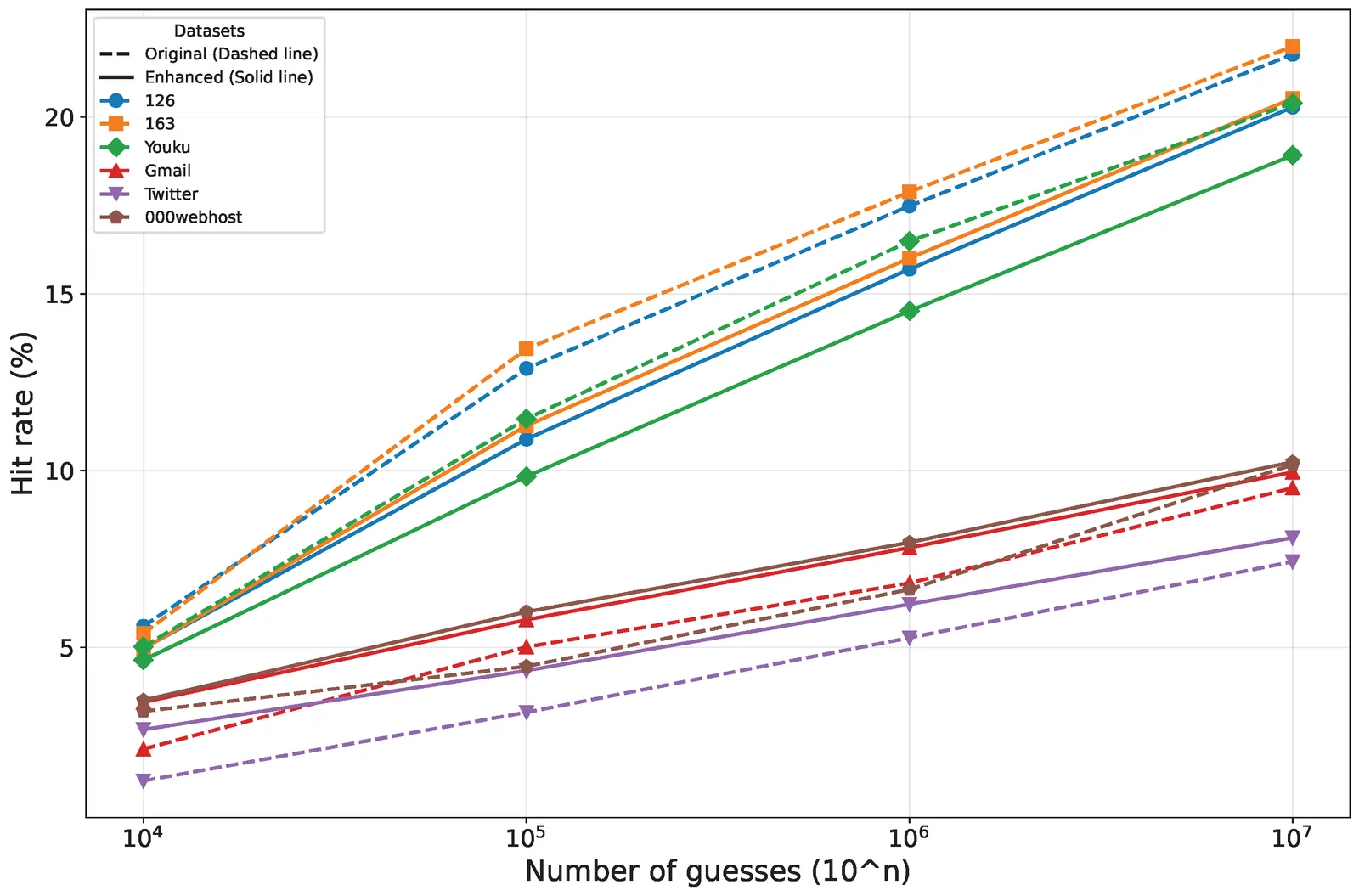
Hit rate of B_CN_test in Chinese environment before and after enhancement.

**Figure 8:**
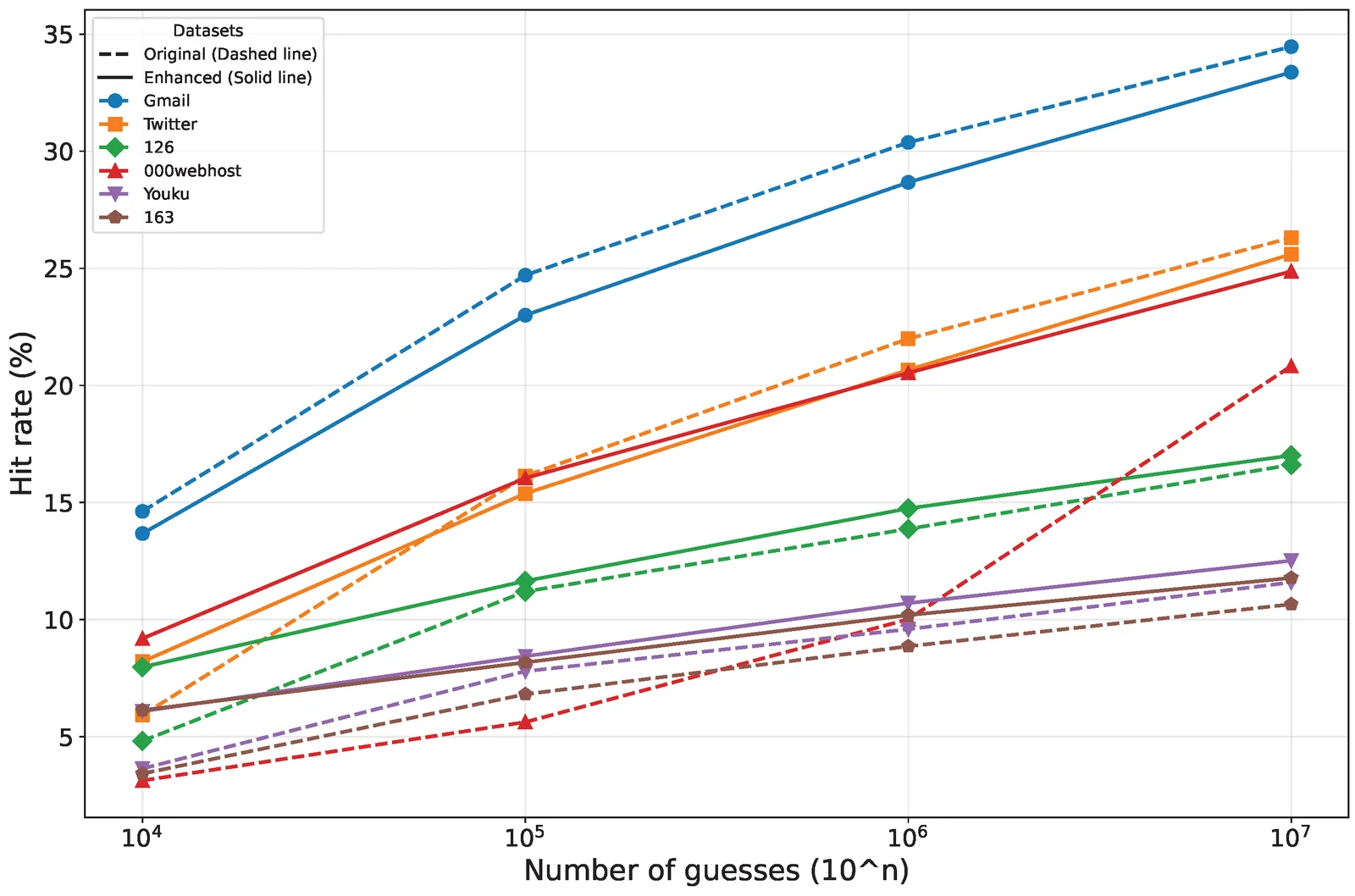
Hit rate of B_EN_test in English environment before and after enhancement.

Using the feature-enhanced probability dictionary, we constructed a more targeted post-guessing dictionary and verified its effectiveness in improving the hit rate with a small-sample test set, denoted as *B*. By comparing the original hit rate to the feature-enhanced hit rate, the results presented in Figure 7 and Figure 8 were obtained.

Among the figures, Figure 7 illustrates the hit rate of $A_{i}$ on B_CN_test before and after feature enhancement in a Chinese environment, and Figure 8 depicts the improvement in hit rate of $A_{i}$ on B_EN_test before and after feature enhancement in an English environment. The horizontal axis of the graphs represents the number of guesses, including values such as $10^{4}$ , $10^{5}$ , $10^{6}$ , and $10^{7}$ , among others. The vertical axis indicates the percentage value of the hit rate. A negative value signifies a decrease in the hit rate, while a positive value indicates an increase. The twelve groups of lines in the graphs represent the hit rates of six sets of password combinations under varying numbers of guesses. Each of the twelve lines corresponds to one of the six password sets, differentiated by color and label. Dashed lines represent the original hit rates, while solid lines denote the enhanced hit rates.

By analyzing the experimental results, it is evident that when the custom parameter α is set to 1, the implementation of the feature enhancement algorithm significantly improves the system’s hit rate performance in low-similarity scenarios, as indicated by the similarity calculations in Experiment 2. Specifically, when the similarity between *B* and $A_{max}$ falls below a threshold of approximately 0.6, the hit rate can be substantially enhanced through the use of feature enhancement, achieving an improvement of up to 10.52% compared to the rate prior to enhancement for $A_{max}$ . Based on these findings, we propose a dynamic training strategy selection mechanism: for high-similarity scenarios with a similarity greater than 0.6, the original $A_{max}$ is utilized directly as the training set; conversely, for low-similarity scenarios with a similarity less than 0.6, $A_{max}$ enhanced with *B* features is preferred as the training set, as detailed in the specific formula in Section 3.3. This strategy ensures optimal performance in high-similarity scenarios while effectively enhancing model performance in low-similarity situations.

## 5. Conclusions

The performance of traditional probabilistic context-free grammar (PCFG) methods in small-sample scenarios is constrained by the size of the password set. To address this challenge, this paper proposes an approach that combines cross-domain similarity measures with a feature enhancement mechanism. This approach aims to improve the effectiveness of password guessing in data-scarce scenarios.

The method specifically generates a probabilistic dictionary by training a set of passwords using the probabilistic context-free grammar (PCFG) model, converting it into a structured vector representation. This approach enables the measurement of similarity at the structural level between different sets of passwords. In Experiment 1, we systematically evaluate the stability of the structured vectors across various sample sizes and find that the similarity computation demonstrates strong consistency and discriminative ability when the sample size reaches 150 or more. Subsequently, we select multiple publicly leaked password sets from both Chinese and English environments, compare their similarity to the target small-sample password set, and rank them according to their similarity as potential training sets. The results of Experiment 2 indicate that the hit rate of the password set with high similarity to the small-sample target can be significantly improved by up to 32% following direct training. Furthermore, by introducing a feature enhancement strategy in Experiment 3, the hit rate can be increased by as much as 10.52% across multiple experimental sets, thereby validating the effectiveness of the enhancement strategy in improving small-sample password modeling.

In summary, the method proposed in this study effectively models and accurately predicts small-sample password sets by utilizing structural similarity and feature enhancement, all without requiring prior knowledge of the target domain. This approach demonstrates strong generalizability and practicality. Future work will focus on further developing the dynamic adjustment mechanism of the enhancement algorithm to better adapt to a wider range of diverse and complex practical application scenarios.

Finally, it is worth noting that the findings of this study can provide system security administrators with more effective password policy configurations to better defend against increasingly sophisticated traversal attacks. Meanwhile, penetration testers can also utilize these results to obtain higher-hit-rate password datasets, thereby enabling more accurate and reliable security assessments of information systems. 

## Figures and Tables

**Table 1:** Data Set.

Name	Sample Content	Training Set	Test Set
**Small Command Set** B			
B_CN	12306	500	69,500
B_CN	12306	300	69,700
B_CN	12306	200	69,800
B_CN	12306	150	69,850
B_CN	12306	50	69,950
B_CN	12306	20	69,980
B_CN	12306	10	69,990
B_EN	cashcraete	500	69,500
B_EN	cashcraete	300	69,700
B_EN	cashcraete	200	69,800
B_EN	cashcraete	150	69,850
B_EN	cashcraete	50	69,950
B_EN	cashcraete	20	69,980
B_EN	cashcraete	10	69,990
**Large-scale Command Cluster**			
$A_{1}$	126	70,000	\
$A_{2}$	163	70,000	\
$A_{3}$	youku	70,000	\
$A_{4}$	Gmail	70,000	\
$A_{5}$	Twitter	70,000	\
$A_{6}$	000webhost	70,000	\

**Table 2:** The similarity between B_CN , B_EN , and $A_{i}$ .

Similarity	B_CN	B_EN
126	0.74	0.29
163	0.72	0.22
youku	0.63	0.24
Gmail	0.52	0.89
Twitter	0.36	0.77
000webhost	0.35	0.25

## Data Availability

The original contributions presented in this study are included in the article. Further inquiries can be directed to the corresponding author.
